# Telemedicine: The legal framework (or the lack of it) in Europe

**DOI:** 10.3205/hta000126

**Published:** 2016-08-16

**Authors:** Vera Lúcia Raposo

**Affiliations:** 1 Faculty of Law of Macao University, Macao, China; 2 Faculty of Law of Coimbra University, Coimbra, Portugal

**Keywords:** telemedicine, privacy, medical malpractice, e-commerce, European Union (EU)

## Abstract

In the framework of European law telemedicine is, simultaneously, a health service and an information service, therefore, both regulations apply. In what concerns healthcare and the practice of medicine there are no uniform regulations at the European level. Concerning health services the most relevant achievement to regulate this domain is Directive 2011/24/EU. In what regards information and telecommunications we must have in consideration Directive 95/46/EU, Directive 2000/31/EC and Directive 2002/58/EC. However, many issues still lack uniform regulation, mainly the domain of medical liability and of medical *leges artis*. Probably such standardization will never take place, since the European Union does not have, until now, a common set of norms regarding tort and criminal liability, much less specific legal norms on medical liability. These gaps may jeopardize a truly European internal market in health services and hamper the development of telemedicine in the European zone.

## 1 The concept of telemedicine

Telemedicine can be defined, according to the European Commission, as ‘the provision of healthcare services, through the use of ICT, in situations where the health professional and the patient (or two health professionals) are not in the same location. It involves secure transmission of medical data and information, through text, sound, images or other forms needed for the prevention, diagnosis, treatment and follow-up of patients’ [1]. 

In the quoted definition the Commission refers to the definition of ‘health professionals’ as it is defined on Article 3/f of Directive 2011/24/EU [2]:

“health professional” means a doctor of medicine, a nurse responsible for general care, a dental practitioner, a midwife or a pharmacist within the meaning of Directive 2005/36/EC, or another professional exercising activities in the healthcare sector which are restricted to a regulated profession as defined in Article 3(1)(a) of Directive 2005/36/EC, or a person considered to be an health professional according to the legislation of the Member State of treatment.

In sum, telemedicine allows medical acts and connected health procedures at a distance, though it does not overrule the need of a personal contact, nor intends to substitute the so-called standard medicine [3], [4], [5]. It covers very different realities [6], [7]. Sometimes there is a real time interaction amongst the participants, while other times there is a time lapse between each intervention. Different devices may be used to communicate at a distance, such as videoconferencing units, e-mail, webcams, PDA or smartphones. The channel used to allow communication may also vary (broadband, network, wireless). In the present study we will refer to telemedicine as a broad concept, encompassing every type of at distance medicine. However, more detailed concepts have been created to define specific realities, namely cybermedicine (the practice of medical acts using internet) and e-health (the interchange of health information by means of new communication technologies). In sum, telemedicine can take many forms, each with its own specificities. In this study we will mainly deal with telemonitoring, tele-education, teleintervention and teleconsultation, all mechanisms to practice medical acts at distance.

Telemonitoring relates to the control of vital signs at a distance, through systems installed away from the doctor and even portable by the patient, that send alarm signals to a remote control centre. This possibility is not insignificant in terms of healthcare costs, to the extent that it allows a cheap provision of medical care without institutionalization. 

Tele-education translates into the provision of general and technical information made accessible to the general population or other health professionals, respectively. The so-called ‘virtual hospitals’ fall into this category, as they constitute a form of information transmission (procedure rules, preventive measures, clinical cases) between health professionals through websites; however, despite its potential it remains a form of knowledge diffusion rarely used in many European countries. More common are websites aimed to provide the general public with information on healthcare, some quite technical and specific, that come to resemble the advice that a doctor would give to his patient; however, those ‘opinions’ are given in general, regardless the specificities of the patient’s clinical situation.

Teleintervention relates to surgical interventions applied at a distance, resorting to mechanics – such as robotized and computerized machines – which allow the physician to intervene on the patient’s body without actually touching it.

Teleconsultation process is similar to the traditional medical consultation one, with the difference that the doctor and the patient are physically separated and communicate at a distance, establishing a real-time conversation through videoconference, phone [8] or chat. This method is likely to be applicable to almost all medical specialties, even the ones requiring the existence of an actual physical examination, as long as the patient is accompanied by another healthcare professional capable of performing the examination and of reporting back the results to the physician who takes care of the remote consultation. In some other cases it is possible to overcome the physical distance using particular technology, for example, by allowing stethoscopes to remotely listen to the heartbeat.

Finally, another embodiment of telemedicine that may also be called teleconsultation refers to contacts between two or more healthcare professionals regarding a certain medical issue. Good examples of this consultation method amongst health professionals are ultrasound transmission mechanisms and other similar transmission apparatus that are used to work between hospitals or between hospitals and ambulances.

## 2 Benefits of telemedicine for European patients and healthcare providers

Telemedicine involves several benefits, both for physicians and medical institutions as well as for patients [1], [9]. That being so it plays a major role for the development of medicine and healthcare delivery in Europe.

In effect, telemedicine allows remote group collaboration between various healthcare professionals from different locations, sometimes even from different countries. Practitioners can communicate with distant colleagues, thus improving the quality of the services provided. The continuous flow of communication between healthcare professionals is motivated by the growing complexity of medicine, which forces doctors to consult with more experienced colleagues or experts in a particular field or just to request a second opinion. All of these behaviours are considered good medical practice (provided that they do not delay an urgent procedure, nor constitute defensive medicine), and actually the absence of such contacts is what may hold the doctor liable for his disinterest and carelessness. On the other hand, patients – that are nowadays more aware of their rights and more alert on the risks of medical faults – also look for second opinions, sometimes from doctors that they have never actually met, by simply using the phone, the e-mail or even a website.

In the perspective of healthcare institutions telemedicine opens the possibility to expand the spectrum of medical services available, thus increasing the chances for profits. 

Furthermore, it may be quite convenient for patients themselves. Telemedicine facilitates patient’s direct access to a distant doctor, without requiring the displacement of any of the participants, allowing access to some forms of medical care that otherwise would not be accessible. In effect, telemedicine may prove to be an important tool in cases in which geographical distance hampers access to healthcare, especially specialised one, as it is the case in many rural areas [10] or in developing countries [11], which lack health infrastructures [12], [13]. Medical treatment in those scenarios would require long travels – not always possible and usually quite expensive, time consuming and exhausting – either for doctors or for patients. Thus, most of the times patients are simply deprived of having access to proper healthcare. Telemedicine may also be a potentially very useful tool in preventive medicine, by providing patients with useful information about their health condition. It can be particularly relevant for controlling chronic conditions (diabetes and heart conditions) – a growing public health issue motivated by the increase on life expectancy and the growing size of the elderly population, though these diseases are also rising in the young population – without keeping the patient in an hospital (telemonitoring) [14]. Not only it is more convenient for the patient, who is able to keep his quotidian life as normal as possible, as it significantly reduces the costs derived from hospitalization and, in the end, the overall healthcare costs, which are a relevant concern in a period of economic crises. On the other hand, it is tempting for those patients that may feel more comfortable to ask questions about sensitive issues (as addictions or venereal diseases) to a distant doctor [14]. Telemedicine also has a great potential regarding patient’s safety, by avoiding some human mistakes. Let’s take as an example what happens with the electronic prescription and how it prevents errors derived from difficulties as to read doctor’s calligraphy [15].

The improvement of healthcare delivery is another important driver. This goal is achievable by several means: the patients’ involvement in their own healthcare issues, which is a mechanism to empower the patient; the increase of patient’s health education and the technical education of healthcare professionals by using new communication technologies; and the easiness to access a second opinion from a specialist, especially important for patients in rural areas or for the ones suffering from orphan diseases.

Despite all these benefits, telemedicine is still far from being widely used in Europe. Difficulties include the huge costs of implementing a telemedicine service [16], hindrances with the interoperability of technical infrastructures among the Member States, concerns about confidentiality and privacy of health data, lack of ethical rules and of *leges artis* specifically applicable to telemedicine, hesitations from health professionals regarding their liability exposures, and especially the dubiety regarding the legal framework of telemedicine in Europe. 

## 3 The legal framework for telemedicine within the European legal order

As a healthcare service, telemedicine is included in the scope of Articles 56 and 57 of the Treaty on the Functioning of the European Union (TFEU), thus, a service, and to that extend is subjected to the general freedom regarding free movement of services.

Nonetheless, this is not the only set of norms applicable to telemedicine within the European legal order.

In fact, in the framework of European law, telemedicine is simultaneously a healthcare service and an information service (a service normally provided for remuneration, remotely and by electronic means at individual request), therefore, both regulations – the ones regarding healthcare and the ones regarding information society services – apply [17].

Concerning information and telecommunications, we must take into consideration the following documents: Directive 95/46/EU [18], the Data Protection Directive, and the forthcoming Data Protection Regulation (GDPR) [19]; Directive 98/34/EC, the Directive on Services of the Information Society [20], [21]; Directive 2000/31/EC, the Electronic Commerce Directive [22]; and Directive 2002/58/EC, the Directive on Privacy and Electronic Communications or e-Privacy Directive [23]. Concerning health services the most relevant achievement to regulate this domain is Directive 2011/24/EU, to so-called Cross-Border Directive [2].

In addition, the European Union (EU) developed several initiatives along the years in order to increase the use of telemedicine in Europe:

- Decision 276/1999/EC of the European Parliament and of the Council of 25 January 1999, adopting a multiannual Community action plan on promoting safer use of the Internet by fighting illegal and harmful content on global networks [24]
- The European Council at Feira, on June 19-20 2000, which supported an initiative within eEurope 2002 to develop a core set of Quality Criteria for Health Related Websites [25], [26]
- The 2002 eEurope strategy, aimed at stimulating secure services, applications and content, based on a widely available broadband infrastructure
- The eHealth Action Plan, adopted in 2004, to support the widespread of information and communication technologies in the health domain, which was afterwards followed by the eHealth Action Plan for 2012-2020
- The Action plan for a European e-Health Area {SEC(2004)539} / COM/2004/0356 final
- The Communication from the Commission to the Council, the European Parliament, the European Economic and Social Committee and the Committee of the Regions making healthcare better for European citizens (COM/2004/0356) [27]
- The Communication from the Commission to the Council, the European Parliament, the European Economic and Social Committee and the Committee of the Regions titled ‘i2010 – A European Information Society for growth and employment’ (COM/2005/0229) [28]
- The 2008-2011 European Patients Smart Open Services project (epSOS) and its related thematic network Calliope to develop and validate cross-border interoperability of patient summaries and ePrescription solutions
- The Commission health strategy (2008-2013) COM(2007) 630 final [29], which provides a framework and objectives to the European work on core health issues, on integrating health in all policies and on addressing global health threats
- The Commission Communication COM/2008/0689 (COM/2008/0689 final) on telemedicine for the benefit of patients, healthcare systems and society [30]
- The Commission Recommendation of 2 July 2008 on cross-border interoperability of electronic health record systems [31] (it provides guidelines for interoperable electronic health record systems, allowing for cross-border exchange of patient data within the Community so far as necessary for a legitimate medical or healthcare purpose)
- The Network of national responsible authorities on eHealth – Commission implementing decision 2011/890/EU of 22 December 2011 providing the rules for the establishment, the management and the functioning of the network of national responsible authorities on eHealth, from 22 December 2011 [32]
- The Communication from the Commission to the European Parliament, the Council, the European Economic and Social Committee and the Committee of the Regions on eHealth Action Plan 2012-2020 – Innovative healthcare for the 21st century [33]
- The Guidelines on minimum/nonexhaustive patient summary dataset for electronic exchange in accordance with the cross-border Directive n. 2011/24/EU, from 19 November 2013 [34]
- The Guidelines on ePrescriptions dataset for electronic exchange under the Cross-Border Directive 2011/24/EU, from 18 November 2014 [35]
- The Green paper on mobile health from 2014, aimed to develop the use of mobile devices to enhance the European’s citizens health [36].

The objective of these initiatives is to turn telemedicine into a standard medical service, accessible to every European patient and fully covered by the respective social security system [10].

Even economically the EU efforts are quite significant, since the EU has invested more than € 500 million in research funding on e-health tools development [37]. 

However, the undeniable value of telemedicine should not conceal its many difficulties, some of them also present in traditional medicine, but which assume a new complexity within telemedicine. For instance, when healthcare professionals from different countries and different idioms collaborate, which idiom should be used to register data in the patient’s health record? Who is in charge of providing technical training (the so called e-literacy) to healthcare professionals using new communications technology? Should all electronic health records (EHRs) be integrated in a common global e-health infrastructure, accessible from every point of the EU?

In face of this vast roll of questions the EU should intervene in a more active way. The fact is that, despite the many working documents and position papers issued by the European institutions, we do still lack a uniform set of norms to regulate telemedicine in Europe. As already referred, a big part of the regulatory competences in these domains still rests with the Member States, who retain most of the competences regarding healthcare and medicine. However, the problem is that the approach to telemedicine varies immensely in between the European countries, even regarding the chosen perspective to regulate telemedicine: some states approached telemedicine from the perspective of laws in the field of IT technologies, while other enacted laws in the domain of healthcare delivery or even in the social security domain. So, even nationally wise there are legal voids that threat the practice of telemedicine, by leaving patients unprotected and healthcare providers fearful of this brave new world.

In the present study we will briefly expose some of the main questions raised by cross-border telemedicine within the European territory. This article restricts its analysis to a particular scenario: cross-border telemedicine within the European territory; therefore, we will not deal with telemedicine activities that exclusively take place in one of the Member States.

## 4 Patient’s rights in cross-border telemedicine in light of Directive 2011/24/EU

For many years European patients were receiving healthcare treatments in other EU countries based on the free circulation of services, firstly guaranteed by the Treaty of the European Community (TCE) and afterwards by the TFEU. The European Communities Court of Justice (Court of Justice) has issued several decisions on this topic – see, for instance, the Watts case [38], concerning a British patient that travelled to France to receive medical treatment and afterwards had the reimbursement denied by the British authorities – but there were still many pending subjects that the Directive 2011/24/EU came to clarify [39], [40], [41], [42], [43].

The Directive (without further specification all articles referred come from Directive 2011/24/EU) covers a large range of issues. One important contribution from this Directive is related with the recognition of prescriptions issued in another Member State (Article 14). The Directive provides special rules on how to identify the medicine prescribed (whose designation may vary amongst Member States) and how to identify the prescriber (Article 11). In addition, it also regulates matters as reimbursement, international cooperation between healthcare entities and cross border enforcement of patients’ rights (namely Articles 3/d, 7/7 and 14), applicable each time telemedicine involves doctors and patients from different countries. So, whenever telemedicine operates beyond national borders the regime of this Directive will apply. On the contrary, whenever telemedicine operates within national boundaries the Directive is not applicable, since the competence to regulate healthcare delivery rests mainly with national states.

In effect, most of the rights guaranteed to patients in cross-border telemedicine come from the Directive 2011/24/EU, which comprises:

1. Right to receive medical treatment in another Member State (Article 1) and, under certain conditions, to be reimbursed (Chapter 3);
2. Right to access one’s medical record, in written or electronic format (Article 4/2/f), containing all the relevant information related with the treatment received;
3. If requested, the patient is also entitled to be informed about the standards and guidelines on quality and safety in place in the Member State where the treatment is provided or, in the case of telemedicine, where the physician is based (Article 4/2/a);
4. Right to be informed on the availability, quality and safety of the service used, as well as information on the authorization or registration status of the teleprovider (Article 4/2/b);
5. Right to have transparent complaint procedures implemented, for patients to seek remedies if they suffer any harm from the treatment received in accordance with the legislation of the Member State of treatment, i.e., where the healthcare provider is located (Article 4/2/c).

The protection of the healthcare provider is also recognized through the implementation of professional liability insurance systems or the guarantee that similar arrangements are in place (Article 4/2/d), a measure that indirectly ends up also beneficiating the patient, by assuring a prompt compensation in case of injury.

The reimbursement of healthcare treatments received in another Member State was, until the Cross Border Directive, a huge difficulty for a free market of medical services. The Directive aimed to solve this problem by establishing, in Article 7, the general rules on reimbursement for cross-border medical acts received [40], [41], [42], [43]. This solution should be concatenated with the national rules regarding public funded health services and the national healthcare service, since healthcare reimbursement falls within the competence of the Member States. Typically patients are asked to pay upfront and are reimbursed afterwards by their home state (though it is also possible to have a national entity paying the healthcare provider abroad directly) on the same amount they would have received in their home country (Article 7/4). Therefore, these rules on reimbursement will only be applicable if the same or an equivalent treatment is available to the patient on his home state, and inasmuch that particular medical act is covered by his country’s national health service if it would take place there, with the derogations pertaining to Regulations 883/2004 [44] and 987/2009 [45]. This is also the solution applicable to telemedicine, although the fact that the patient does not have to move drastically diminishes the costs involved.

However, this solution may be limited or modified according to special circumstances. 

For instance, the national state may pay just a part of the treatment, instead of providing total reimbursement, grounded on ‘overriding reasons of general interest’ (Article 7/9), that are basically related with the need to control the national budget, to avoid unnecessary expenses and to avoid resources waste.

Another exception to the rules on reimbursement comes from Article 8, which allows the introduction of a system of prior authorization for the reimbursement of medical expenses [42]. Prior authorization can only be demanded for treatments that require at least one hospitalization night, or require specialized and cost-intensive medical treatment. This last safeguard can be easily applied to telemedicine, because of the complex – and eventually very expensive – technologies involved. Prior authorization may be refused when grounded in objective reasons, for instance if the patient will be exposed to an excessive risk, which may be a frequent scenario in telemedicine whenever we face a medical act not apposite to be practised at a distance (actually, this would be a good example of a situation in which the doctor should not resort to telemedicine at all).

Furthermore, reimbursement can simply be denied if the home state considers that the treatment in question could have been adequately provided internally, for general interest reasons or even in cases in which prior authorization was required but not requested (Article 8). 

All limitations imposed to reimbursement must be necessary and proportionate and can never operate as discriminatory mechanisms or as an obstacle to the free circulation freedoms typical of European law.

Nonetheless, telemedicine may be a potential target for some of the referred limitations on reimbursement. For instance, one of the situations where previous authorization must be requested is when there are serious concerns based on the safety and quality of the healthcare provided, which may become a frequent scenario in telemedicine because of the risks (supposedly) associated with this practice. The way to override this possible obstacle is to create specific European rules on telemedicine, since the referred restriction does not apply to healthcare practices that have been subjected to harmonization. The reason is that European rules are supposed to impose a minimum level of safety and quality; therefore, those rules would ground legitimate expectations as to the safety of telemedicine.

## 5 Licensing and qualifications: Can Directive 2005/36/EC be applicable?

In the context of healthcare services the topics of qualification, registration and licensing for healthcare professionals have been largely discussed.

Since telemedicine may involve doctors providing healthcare services in a location different from the patient’s home country, it is necessary to define which national legal order (or legal orders) is going to define those requisites. The EU has issued a Directive on recognition of professional qualifications for regulated professions, the Directive 2005/36/EC [46]. However, its provisions do not apply here since they ‘shall only apply where the service provider moves to the territory of the host Member State to pursue, on a temporary and occasional basis’ the activity in question (Article 5/2 of Directive 2005/36/EC). But in telemedicine the service provider does not move to another host state, thus, solely the requisites of his home country (i.e., where he is located) apply. Therefore, is it up to each national entity in charge of regulating the medical practice and the delivery of healthcare services to regulate the qualifications and other legal and/or deontological aspects of every healthcare provider based on its territory, even the ones involved in cross boarder telemedicine (country of origin principle). Apart from those, no other requisite can be imposed on health professionals, not even the ones demanded by the legal order in force where the medical act is received, nor can the doctor be required to obtain any authorization or license there. The Commission Staff Working Document on the applicability of the existing EU legal framework to telemedicine services clarifies that a healthcare professional offering telemedicine needs only to be registered in the country where he/she is physically established, as defined by the E-Commerce Directive (2000/31/EC) [22] and the Cross-Border Healthcare Directive (2011/24/EU) [2].

However, the demanding of a specific licence could be seen as an interesting feature, not because it is a cross-border medical act, but because it is a telemedical act. This idea has already been suggested by some authors [47], demanding the passing of a test that would cover issues specifically related with telemedicine expertise, namely the ability to cope with the new technologies involved. 

## 6 Protection of personal data in telemedicine: the Data Privacy Directive

### 6.1 European regime on health data privacy

Telemedicine involves the circulation of very sensitive data – the patient’s health information –, which is considered personal information by the European law; thus, the EU imposes particular requirements regarding health data protection.

The Directive 2011/24/EU establishes, in Article 14, a voluntary network for cooperation between the national authorities responsible for e-health in each Member State, but this purpose must take into consideration the remaining European legislation in this domain, namely the Data Protection Directive, the future GDPR and the E-Privacy Directive [7], [16], [48], [49], [50], [51]. The aim of these regulations is, on the one hand to allow private data circulation, and on the other hand to adequately protect the data holder.

Personal data are defined as:

any information relating to an identified or identifiable natural person (‘data subject’); an identifiable person is one who can be identified, directly or indirectly, in particular by reference to an identification number or to one or more factors specific to his physical, physiological, mental, economic, cultural or social identity. (Article 2/a of Data Protection Directive). 

Or, in a more simplified way, ‘any information relating to a data subject’ (Article 4(2) of GDPR).

Some data are considered particularly sensitive because they reveal intimate information about the person, such as data concerning health or sex life (Article 8/1 of Data Protection Directive and Article 9/1 of GDPR). That kind of data can only be processed if some legally stated conditions are fulfilled.

Usually the person’s consent is required. However, sensitive data processing can still be licit, even without consent, in some particular contexts, as for instance ‘preventive medicine, medical diagnosis, the provision of care or treatment or the management of health-care services’ (Article 8/3 of Data Protection Directive and Article 9/2 of GDPR).

In order to have the patient’s consent it is necessary to provide him with all the necessary information: the purpose of the data gathered (diagnosis, treatment); the fact that that data are going to be disclosed to a third party (namely, another practitioner); and if the data are going to be send out of the EU territory (for instance, to a data centre in another continent). Furthermore, the patient should always be aware that, no matter how many safety measures are implemented, their data are not completely safe. Having all that into consideration, the patient is required to consent on the handling of the data [49]. According with Article 2/h of the Data Protection Directive (an idea in some way repeated by Article 7 of GDPR), 

the data subject’s consent shall mean any freely given [i.e., with no coercion] specific [i.e., for a specified purpose] and informed indication of his wishes by which the data subject signifies his agreement to personal data relating to him being processed. 

The burden of proof regarding the existence of the consent falls on the controller, but it is easy to demonstrate, as consent must be given in written form and must be autonomized for the remaining content of the document (Article 7 of GDPR).

Even when data processing relies on the patient’s consent, some other requisites must be fulfilled. 

The amount of collected data should be guided by the principle of proportionality. That is, on the one hand the data collectors must have sufficient data resources to provide the healthcare service in an efficient way; but, on the other hand, they cannot collect more data than the necessary for the efficient performance of the medical act.

A concern to have in mind is to guarantee that the collected data are used solely for this purpose and not for any other subsequent purpose. Namely, data cannot be commercialized (it is forbidden to sell data to insurance companies or to marketing companies) in order to obtain profit from personal information.

It must be secured to the patient access to a copy of all data concerning himself, namely his health record, either a paper record or – as it is more common nowadays and it is especially adequate to telemedicine – an electronic health record [52].

Another condition imposed for the licit processing of data imposed is that these data must be processed by a healthcare professional, bound by a legal commitment of professional secrecy. However, that does not always happen, since IT operators, who are only bounded to secrecy by means of their work contract, frequently handle sensitive data.

Most of the obligations regarding data protection rest with the so-called ‘controller’. However, other entities may collaborate on data processing, such as cloud storage suppliers [53] or Internet services providers, which may be considered subcontractors for this purpose (although it may also happen that the controller takes care of the whole process). The responsibility for failures in data processing fits, *prima facie*, the controller, who is in charge of selecting a subcontractor that provides adequate guarantees of security and reliability, therefore, the controller may be held liable if the subcontractor is chosen with poor care and diligence. The allocation of responsibilities amongst the various participants can become very complex and 

the first and foremost role of the concept of controller is to determine who shall be responsible for compliance with data protection rules, and how data subjects can exercise the rights in practice. In other words: to allocate responsibility [53]. 

The adequacy of the level of protection will be evaluated having in consideration the circumstances of the case, namely the purpose of the transference and its duration, the country of origin and the country of destination, the legal norms applicable in the third country and the security measures in place there [48].

In spite of the transposition of this basic legal regime to every Member State, the fact remains that each national legislator has transposed it in a slightly different way. Even the interpretation of the same legal rules by each national entity in charge (usually a national commission for data protection) is quite different, some being very restrictive, while others are much more flexible. Therefore, notwithstanding the common core, it may happen that a certain practice is acceptable in a Member State but not in another, which may raise some legal issues regarding cross-border telemedicine.

### 6.2 Privacy regarding telemedicine

Some of the demands imposed by the regulation on data protection may compromise the practise of telemedicine.

For instance, the Data Protection Directive is especially demanding in what concerns cross borders data sharing, particularly for countries outside the EU, whereas telemedicine frequently requires this kind of data flow. If the data are transferred outside the European Economic Area (EEA) additional restrictions should be considered and the transmitting entity must ensure that the non-Member State provides an “adequate level of protection” (Article 25 of Data Protection Directive and Article 41 of GDPR). For instance, some data movement may require specific contracts between the European operator and the trans-European one [16] or the implementation of specific guidelines. Because the U.S. are a frequent country of destiny for European data, some guidelines were created in order to facilitate and expedite the process, the Safe Harbour Principles Commission Decision 2000/520/EC [48].

The evaluation of the protection effectiveness provided to data by the States outside EU will take into account the circumstances of the case, namely the purpose of the transference and its duration, the country of origin and the country of destination, the legal norms applicable in the third country and the security measures in place there [48]. To facilitate the cross-border data flow it is recommendable to implement a prompt patient’s consent procedure, accepted in every European legal order [10].

A problem may arise due to the frequent use of cloud databases to storage data in telemedicine, since this note may difficult the compliance with the demanding regime imposed by the Data Protection Directive.

Another possible obstacle imposed by the data protection regulation comes from the fact that access to health data is usually limited to health professionals bound by the professional obligation of secrecy, while telemedicine usually involves IT staff, who also have access to, at least, some of that data. As above mentioned, in order to solve this difficulty it is recommendable to issue a more flexible regulation, allowing the access to health data by IT technicians provided they sign a contractual commitment of confidentiality [10]. 

### 6.3 Safety measure to guarantee compliance with privacy requirements 

The existing regulation devotes close attention to the safeguard of the collected data; thus, impose severe sanctions for the unlawful or/and unauthorized disclosure of data, their loss or destruction, and to any privacy breach. Therefore, the data handler (the company in charge of data handling, the hospital or even the doctor, depending on the specific contractual relations between those participants) is obliged to take appropriate measures in order to guarantee data safety, namely the use of reliable encryption techniques or of potent firewalls. 

Some of the most dangerous vulnerability causes in the context of telemedicine within a hospital relate to poor encryption, flaws in the process of separating internal networks from external networks, lack of inventories regarding computer devices and other equipment authorized to access the hospital network, deficiencies in user’s authentication, and inability to identify intruder devices on the wireless network [54]. In order to avoid liability issues it may prove useful to implement solutions as data encryption, closed networks and electronic signatures, all of them valuable tools aimed to maintain the confidentiality, integrity and authenticity of the transmitted health data [7].

A very common form of telemedicine operates using mobile health applications (apps) [55] and electronic devices. Medical applications and devices that support telemedicine services must comply with multiple privacy and security rules, for instance, regarding GPS location. They must also comply with rules regarding producer liability [56] and even, depending on the specific kind of product, with rules regarding medical devices [57] being that all of these are very strict norms in the European context.

In the context of apps and websites a concept that became quite frequent is the one of privacy by design, which intends to express the connection between the conceptualization of these information society instruments and the various concerns related to privacy [58]. In order to promote this particular feature – privacy by design – many privacy-enhancing technologies (PETs) have been developed and flourished in the last decade, aimed to design information and communication systems in such a way that they operate using less data and, simultaneously, comply with the legal requirements applicable [59], [60]. 

## 7 Telemedicine services as a form of e-commerce: the E-Commerce Directive

Since telemedicine is a service frequently provided by Internet, it falls under Directive 2000/31/EC [61]. Note that the definition of ‘information society services’ – that comes from Directive 98/34/EC and from Directive 98/84/EC [62] – covers any normally remunerated kind of service, provided at a distance, per electronic equipment for data processing (including digital compression) and for data storage, at the individual request of the service recipient. Therefore, every healthcare service supplied at distance for any kind of fee falls within the scope of this Directive.

Within this Directive some norms are particularly important in what regards to telemedicine. Article 3 established the country of origin principle [63], i.e., the service provider has to comply with the country of the commercial establishment’s legal requirements, not with the legal order in place where the service is received (although with some exceptions in Article 3/3 of Directive 2000/31/EC). According with Article 5, there is a minimum of information to be provided to the service receiver. Article 8, which deals with regulated professions – as it is the case of healthcare professionals – assigns Member States with the task of controlling if healthcare professionals are respecting their professional rules when offering information society services and also encourages the creation of an European Good Practices Guide ‘in order to determine the types of information that can be given for the purposes of commercial communication’ (Article 8 of Directive 2000/31/EC).

## 8 Difficulties in defining an European standard of care for telemedicine

Telemedicine – especially when it operates cross borders – is still a new reality in Europe. Patients don’t trust telemedicine services enough yet, probably because the applicable legal regulations are insufficient and the existing ones are not clear.

In addition, the standard of care for healthcare providers operating in telemedicine becomes more demanding, therefore, requiring special considerations and probably a common standard delineation – though a basic one – in the European legal order.

However, there are no European norms dealing with the substantial regime of medical liability (or, as a matter of fact, with tort or criminal liability in general terms), nor with the standard of care for healthcare providers. The only competences that the Treaty on the Functioning of the European Union (TFEU) assigns to the EU regarding health issues relates with public health (Articles 4/2/k and 168 TFEU, though Article 168 TFEU assigns the responsibility for organizing and delivering health care to Member States, while the EU only holds limited competences in this regard) and questions connected with the four fundamental freedoms of the internal market, as for instance the patients or physicians freedom of movement; while all the remaining issues are of each Member State exclusive responsibility. However, looking closely into Articles 6/a and 9 of TFEU, according to which the protection and improvement of human health is one of the purposes of the EU, this statement seems to embrace a broader perspective on the goals of the EU regarding health issues. In addition, Article 114(3) of TFEU states that the EU must provide due protection to the European citizen’s health as part of the EU’s goal to ensure the internal market functioning.

The EU has been enlarging its competences in this domain with both the European treaties’ successive revisions and the contributions of the Court of Justice [64], so nowadays many health issues are under the competences shared by the EU and the Member States. Many questions entered the EU jurisdiction competence scope because medical treatments were qualified as services according to Article 56 of TFEU. Nevertheless, the legitimacy of the EU to regulate healthcare delivery remains limited and subsidiary.

In sum, the fact that telemedicine may potentially involve so many countries, and does actually comprise so many different domains (medicine, IT, privacy, social security), makes it difficult to define the most adequate entity to regulate and control it. This lack of regulation and authority leaves European patients with some mistrust regarding the legal consequences of a treatment provided through telemedicine.

Harmonization in this regard is particularly difficult having in mind that we are basically dealing with the structure of legal liability in tort and in criminal law. By its own nature such rules differ enormously amongst the European Member States, namely between the continental law systems and the common law systems. Though some basic principles remain the same, some others are quite different (as it happens with the understating of culpability) and even the legal terminology may differ. In addition, within the very same legal order rules about medical liability may differ according with the context of the medical act, i.e., if it is practised in the national health service or in the private sector [65], since tort law is used in the first case and contract law in the second hypothesis (and also varies in a doctor-patient relationship, in a hospital-patient relationship and in a doctor-doctor relationship).

In the case of medical liability we must also have in mind the medical *leges artis* influence on the content of legal rules. Although those *leges artis* do not differ much from legal order to legal order, they still may suffer some geographical particularities, namely dictated by the dominant ethical values. Differences are particularly noticed regarding questions like informed consent or end of life decisions, matters that are treated in light of different ethical and legal rules amongst European Member States, grounded on distinct ethical and philosophical values. Nevertheless, some of the differences amongst Member States do not have an ethical nature. For instance, see the question of anonymous medical services, whose provision is allowed in some Member States (Portugal, Spain, United Kingdom), but forbidden in some others (Finland, Italy) [61]. In addition, certain kinds of health professionals cannot count on a specific regulation all across Europe, as it happens with osteopaths, whom are regulated professionals only in seven European countries, a note that hampers the exact apprehension of the concepts of ‘health care professional’ and ‘health care provider’ as referred to on the Cross-border Healthcare Directive (although Article 3/f of this Directive remits to Article 3(1)(a) of Directive 2005/36/EC and to any person ‘considered to be an health professional according to the legislation of the Member State of treatment’). These legal discrepancies between Member States may raise relevant issues in cross-border healthcare. 

In addition, telemedicine presents particularities derived from its own nature. Admittedly, the rules on tort and criminal liability will be the same ones applicable to standard medicine; however, telemedicine may pose some challenges that must be taken into account. For instance, because telemedicine requires the handling of complex technologies (machines to measure at distance the level of blood sugar or body mass, electronic health records, robots to perform surgical interventions at distance, on-line prescription of drugs), it may demand some specific qualifications from the healthcare professional, not required in conventional medicine.

Many European countries still lack a specific regulation on telemedicine (although some other have already issued their regulations, as for instance the French Décret n° 2010-1229 du 19 octobre 2010 relatif à la télémédecine [66]) and even when it does exist, the regulation is basically concerned with general impositions and not with specific standards of care, which should preferably be issued by a professional and scientific association and not by the lawmaker. Some rules and guidelines have actually been issued. For instance, European Telehealth has created the European Code of Practice for Telehealth Services [67], which states an accreditation by DNV GL (an international certification body and classification society which has as main expertise field technical assessment, research, advisory, and risk management) and an ISO certification. Some professional associations also intervene in this domain issuing some (very) basic guidelines [68].

But the fact is that presently the EU has not yet created a harmonized set of rules regarding tort and criminal medical liability, nor is it conceivable that it will happen while the distribution of competences remains the same. Nevertheless, on the beginning 90s (last century’s, that is) the EU proposed a Directive dealing with the healthcare providers’ liability (OJ C 12/8, 1991), but it was not approved and no other proposal of that kind has been submitted [69]. Therefore, it is up to each Member State to regulate this issue, just as it is up to each Member State to define the qualifications and requirements to practice medicine and consequently telemedicine. According with a Staff Document of the European Commission [70] an healthcare professional operating in telemedicine only needs to be registered in the country where he is established and to comply with the requirements imposed in that legal order. But even the most basic questions, such as the very meaning of what is a ‘medical act’, has proven to be difficult, deserving different definitions from each national legal order (some legal orders do not even hold a definition for medical act, such as the Portuguese one ).

## 9 The future of telemedicine in Europe

The benefits of telemedicine are very seducing. Firstly, and above all, for the patient, since it allows access to medical care that otherwise would not be accessible to him. Secondly, for doctors, inasmuch as telemedicine prevents unnecessary displacements, often quite lengthy and tiring. But also for health institutions, since it may increase their profit by reaching more patients and expanding the spectrum of services available.

In sum, telemedicine allows various distant relationships: on the one hand, the collaboration between several healthcare professionals from different locations, sometimes even from different countries; on the other hand, it simplifies the patient’s direct access to doctors practising in another town or country. Both purposes are achieved without requiring any of the participants to travel, through the use of different telecommunication channels and mobile technologies. 

Nonetheless, many telemedicine features may become problematic, namely privacy breaches, physical distance itself, inclusion of new technological methods and the weakening of doctor/patient relationship. Therefore, healthcare providers should take into account that telemedicine might lead them to face additional potential medical faults within a standard of care that, in some cases, may be substantially more demanding. 

Up until now the EU has still not issued specific rules on medical liability, despite its specificities, and this gap may jeopardize telemedicine’s development in Europe, thus, denying European patients of all its benefits. However, the fact is that the EU won’t be able to create an uniform regulation covering all aspects of telemedicine, but only regarding the ones connected to technology and privacy, which actually are domains already uniformed under European law. As for patient’s rights in the framework of telemedicine, most probably the EU will only be able to create some basic guidelines, in a similar way to what happens to patient’s rights in the context of cross-border healthcare, as results from Directive 2011/24/EU. To go more in details would be problematic because until the present moment the rights of European patient’s still lack a uniform set of rules and to start the uniformization by such a specific and complex domain as telemedicine won’t be a wise option. For a similar reason are not to be expected harmonised rules in what regards medical liability resulting from telemedicine. In effect, and as stated above, Member States have very different national laws to deal with liabilities, namely because the EU congregates continental law models and common law models. Due to this intrinsic difference, any attempt to harmonize tort liability, and especially criminal liability, will be doomed to fail.

In sum, due to several disparities amongst the European legal orders and to the consequent difficulties in drafting a common framework for many aspects nuclear to telemedicine, the EU can only aspire to create a legal framework for those domains in which European law had already had any kind of inventions and adapt it to the specificities of telemedicine; for instance, web communication in telemedicine, privacy in telemedicine, consumer protection in telemedicine. All the remaining issues will continue excluded from EU harmonization and we do not envisage a different solution in the near future. Therefore, it would be up to each Member State to provide a legal framework for telemedicine, while the role of the EU would be limited to require from Member States the obligation to regulate it. 

## Notes

### Competing interests

The author declares that she has no competing interests.
